# Optimizing pediatric venipuncture: ensuring successful blood sample collection with minimal stress and pain

**DOI:** 10.1186/1710-1492-10-S2-A59

**Published:** 2014-12-18

**Authors:** Mary Ann Mauro, Linda Warner, Robby Mamonluk, Stuart E Turvey

**Affiliations:** 1 Department of Pediatrics, Child & Family Research Institute, University of British Columbia, Vancouver, British Columbia, V5Z 4H4, Canada

## Background

The Canadian Healthy Infant Longitudinal Development (CHILD) study is a multicenter prospective birth cohort study designed to examine genetic and environmental factors related to the development of childhood asthma, allergies, and atopic dermatitis. The CHILD study events include the collection of blood samples at ages 1 and 5 years for all subjects. Blood collection is a critical aspect of the CHILD study, and all the following factors require thoughtful consideration: (a) optimizing the success rate of blood collection to ensure scientific integrity; (b) recognizing and relieving stress and pain during the pediatric venipuncture process; and (c) retaining subjects in this longitudinal study.

## Methods

At the Vancouver site of the CHILD study, we reviewed and compared current topical anesthetic creams used for pediatric venipuncture, specifically Ametop Gel (tetracaine hyrdrochloride gel 4%) versus EMLA (eutectic mixture of lidocaine 2.5% and prilocaine 2.5%). We conducted regular in-service training to ensure all members of the study team were informed and educated in understanding the importance of collecting the blood sample and dealing with pediatric populations at different developmental stages. Training also addressed parental anxiety related to the procedure and strategies to establish rapport and trust were shared. Finally, we developed a repertoire of comfort and distraction techniques to support the child, the parent, and the phlebotomist. We used quantitative and qualitative approaches to assess outcomes, including the number of blood samples successfully collected at the 1 and 5 year clinic visits and analysis of responses to our parent satisfaction survey evaluating perceptions of the venipuncture procedure.

## Results

Based on our analysis, we adopted the topical anaesthetic, Ametop Gel (tetracaine hyrdrochloride gel 4%). Rationale for this choice included: vasodilation vs. vasoconstriction, timing of onset, cost effectiveness, and previous experience of team members. Staff training demonstrated consistent ability to prepare and review process with parents before the blood collection event, evident in the number of blood samples successfully collected (35/40 successful collections at the 5 year visits of the pilot vanguard cohort, 87.5%). Parental survey responses indicated satisfaction with use of the topical anesthetic and their child’s comfort with procedure.

## Conclusion

Minimizing stress and pain during the collection of blood samples in research studies is important to the child’s comfort, parental satisfaction, and subject retention. These factors are critical to the success of any longitudinal study. Our detailed and methodical approach to the planning and execution of venipuncture at the Vancouver site of the CHILD study resulted in greater than 85% success rate in obtaining blood samples at the 1 year clinic visit while achieving high levels of parental satisfaction.

